# Single-cell transcriptomics reveals a role for pancreatic duct cells as potential mediators of inflammation in diabetes mellitus

**DOI:** 10.3389/fimmu.2024.1381319

**Published:** 2024-04-29

**Authors:** Amadeo Muñoz García, Juri Juksar, Nathalie Groen, Arnaud Zaldumbide, Eelco de Koning, Françoise Carlotti

**Affiliations:** ^1^Department of Internal Medicine, Leiden University Medical Center, Leiden, Netherlands; ^2^Department of Cell and Chemical Biology, Leiden University Medical Center, Leiden, Netherlands

**Keywords:** diabetes, inflammation, single-cell RNAseq, duct cells, beta cells

## Abstract

**Introduction:**

Inflammation of the pancreas contributes to the development of diabetes mellitus. Although it is well-accepted that local inflammation leads to a progressive loss of functional beta cell mass that eventually causes the onset of the disease, the development of islet inflammation remains unclear.

**Methods:**

Here, we used single-cell RNA sequencing to explore the cell type-specific molecular response of primary human pancreatic cells exposed to an inflammatory environment.

**Results:**

We identified a duct subpopulation presenting a unique proinflammatory signature among all pancreatic cell types.

**Discussion:**

Overall, the findings of this study point towards a role for duct cells in the propagation of islet inflammation, and in immune cell recruitment and activation, which are key steps in the pathophysiology of diabetes mellitus.

## Introduction

Inflammation is a key component in the development of diabetes mellitus, as it contributes to the loss of functional pancreatic beta cell mass that eventually causes hyperglycemia. Type 2 diabetes (T2D) is strongly associated with overnutrition and a sedentary lifestyle that leads to insulin resistance in peripheral tissues. This causes pancreatic beta cells to overcompensate the production of insulin, causing an increase in beta cell stress that may compromise their viability and function. It is well-established that the progression of the disease is accompanied by an increase in inflammation-related processes in both adipose tissue and pancreatic islets (1). Type 1 diabetes (T1D) is an autoimmune disease characterized by a dysregulation of the immune system that, promoted by cellular and molecular immune-related events, generates a chronic pro-inflammatory niche in the pancreatic islets that, ultimately, results in the specific attack and destruction of the beta cells. There is evidence that the defective molecular processes of immune cell tolerance lead to the appearance of auto-reactive T cells from the adaptive immune system, which then act as a direct effector in beta cell destruction. In addition, innate immune cells and pro-inflammatory cytokines infiltrate the pancreatic islets, leading ultimately to the activation of auto-immunity (2). These interactions between the immune system and pancreatic islet cells generate higher levels of pro-inflammatory molecules within the islet that promote what is known as islet inflammation. In addition to T2D and T1D, a rare genetic form of diabetes mellitus, Wolfram syndrome, is caused by increased endoplasmic reticulum stress (ER stress). ER stress-mediated inflammation is strongly associated with beta cell failure and death (3, 4).

*In vitro*, modelling of pancreatic islet inflammation is commonly done by treating beta cell lines or (murine) islets with pro-inflammatory cytokines, including IL1β and IFNγ (5). In addition, IFNα has been reported to be a key molecular signaling component to promote the onset of type 1 diabetes (6, 7). Traditionally, beta cells were the focus for studying the molecular mechanisms underlying inflammation-induced beta cell failure and death (8–13). However, it is unclear how other pancreatic cell types, especially exocrine cells, respond to inflammation, and may secondarily affect islet cells.

Here we used single-cell RNA sequencing (scRNAseq) to explore the cell type-specific molecular response of primary human pancreatic cells to an inflammatory environment that is associated with the development of diabetes mellitus.

## Materials and methods

### Human islets and islet-depleted tissue

Human islets were isolated from the pancreas (cadaveric donors) obtained through the Eurotransplant multiorgan donation program. Islets were used for research only if they could not be used for clinical purposes, and if research consent was present, according to Dutch national laws.

Cells from islets isolated from donors without diabetes (n= 6), and digested pancreatic tissue from donors with a history of T1D (n=2), or diagnosed with Wolfram-syndrome (n=1) were processed for scRNAseq, as well as cells from islet-depleted tissue (pancreatic exocrine tissue left over post islet isolation) obtained from additional non-diabetic donors (n=4).

Donor information related to the samples used in this study can be found in **Supplementary Table 1**.

Islets were cultured in CMRL 1066 medium with 5.5 mmol/L of glucose, 10% fetal calf serum, 20mg/mL ciprofloxacin, 50 mg/mL gentamycin, 2mmol/L L-glutamine, 10mmol/L HEPES and 1.2 mg/mL nicotinamide. Islets and islet-depleted tissue were cultured at 37°C in a 5% CO2-humidified atmosphere. Medium was refreshed upon receipt and every 2 days thereafter. The islet-depleted tissue was kept in its supplemented CMRL 1066 medium, then washed and stored overnight at 4°C in advanced DMEM/F12 with trypsin inhibitor (100ug/ml) before processing immediately in the morning.

### Treatments

Isolated human islets from donors without diabetes (n=3) were exposed to 1 ng/ml IL-1β (401-ML, R&D systems) + 50 ng/ml IFNγ (285-IF, R&D systems), 200 ng/mL CXCL8 (208-IL, R&D systems) or 2000 U/ml IFNα (IF007, Merck Millipore), or left untreated for 24h or 72h. Concentrations were selected based on previous work performed in pancreatic islets treated with cytokines (11, 14). To block CXCL8 signaling under cytokine stress (IL-1β+IFNγ), islets were treated with an anti-CXCL8 blocking antibody (250ng/mL) (MAB208, R&D systems) for 72h.

### Glucose-stimulated insulin secretion test

Human islets (~30 IEQ) per well were placed in a 96-well transwell plate. Islets were preincubated for 90 minutes in low (1.67 mmol/L) glucose-containing buffer solution at pH 7.4 containing 11.5 mmol/L NaCl, 0.5 mmol/L KCl, 2.4 mmol/L NaHCO_3_, 2.2 mmol/L CaCl_2_, 1 mmol/L MgCl_2_, 20 mmol/L HEPES, and 0.2% human serum albumin. Islets were subsequently transferred to low (1.67 mmol/L) glucose-containing buffer solution for 60 minutes and then to high (20 mmol/L) glucose-containing buffer solution for 60 minutes. Insulin secretion was assessed using a human insulin ELISA kit (Mercodia).

### Single-cell transcriptomics

#### scRNAseq - SORT-seq platform

Pancreatic islets were dispersed into single-cells by incubation with 0.025% trypsin (Gibco) supplemented with 10 mg/mL DNase (Pulmozyme, Genentech) for 6–7 min and filtered through a 40 μm cell-strainer to remove undigested material. Briefly, pancreatic islet-depleted tissue was first dispersed mechanically by passing it through a 21G needle, followed by three washes with Hank’s Balanced Salt Solution (Thermo Fisher Scientific) supplemented with 100μg/ml trypsin inhibitor (Merck) at 200g for 4 minutes at 4°C. Tissue was then dissociated at 37°C in a shaker for 15-30 minutes depending on the sample. Dissociation was stopped with Advanced DMEM/F12 (Thermo Fisher Scientific) supplemented with 10% Fetal Bovine Serum (FBS; Greiner Bio-One). The cells were centrifuged at 200g for 3 minutes, at 4°C to collect the cell pellet and resuspended in DPBS (no calcium, no magnesium; Thermo Fisher Scientific) supplemented with 2% FBS and 1mM EDTA (Thermo Fisher Scientific). The cells were then passed through a 40μm cell-strainer.

Single-cell RNA-seq dataset from dispersed cells from islets and islet-depleted tissue was obtained by the SORT-seq technique as Muraro et al. described (15). Cells were FACS sorted into 384-well cell capture plates. Cells were lysed by heat (65 Celsius degrees) followed by a second-strand cDNA synthesis. For each plate, all barcoded material was amplified by *in vitro* transcription. Cel-Seq2 workflow was followed to generate the cDNA library using TruSeq small RNA primers (Illumina). The DNA library was paired-end sequenced using Illumina Nextseq 500, high output, with a 1x75 bp Illumina kit (read 1: 26 cycles, index read: 6 cycles, read 2: 60 cycles).

#### scRNAseq - 10X genomics platform

To investigate the effect of inflammation of pancreatic cells, we explored part of a dataset we partially previously described earlier ((16); GSE218316), to focus specifically on the two pro-inflammatory conditions (IL1β+IFNγ) and IFNα for the current study. This *in vitro* model and Wolfram-syndrome patient datasets were generated using the 10X-Genomics protocol. Islets exposed to the different treatments were multiplexed per timepoint and donor using cell hashing (17). Briefly, each treated islet cell suspension was incubated with a pool of antibodies directed against B2M and CD298 (BioLegend). Thereafter, labeled cell suspensions were pooled per donor and time point and processed for single-cell mRNA sequencing according to standard 10X Genomics 3’ V3 chemistry protocol. Before loading the 10x Chromium controller, the pooled cell suspensions were counted to assess cell integrity and concentration. Cells were loaded and the resulting sequencing libraries were prepared following standard 10x Genomics protocol. The DNA libraries were paired-end sequenced on the NovaSeq6000 platform with v1 and a 300-cycle Illumina kit with a sequencing depth of 25,000 reads per cell (gene expression) and 5000 reads per cell (hashtag oligos). BCL files resulting from sequencing were transformed to FASTQ files with 10x Genomics Cell Ranger mkfastq. FASTQ files were mapped with Cell Ranger count. During sequencing, 28 bp read 1 was used for the identification of the Illumina library barcode, cell barcode, and UMI. Read 2 was used to map to the reference transcriptome GRCh38. Filtering of empty barcodes was done in Cell Ranger. As no significant differences was seen between the two timepoints (data not shown), we merged the data from both time points for this study.

#### Data filtering and processing

Data was processed with the R Seurat package (R version 4.3.0). Cells containing at least 2000 UMI’s with no more than 40% of mitochondrial genes were considered for downstream analysis. Feature expression data was normalized for each cell using the total expression then normalized data was log-scale by a 10,000 factor. When datasets were integrated, data was corrected for the donor batch effect. Finally, to study the Fasolino et al dataset, only cells that had between 200 and 2500 unique feature counts were included in the analysis.

#### Clustering, visualization, and cell type annotation

The top 2000 most variable genes were used for dimensionality reduction and clustering. To identify the dimensionality of each dataset, the top 2000 most variable genes were used. For cell clustering was performed by graph-based method from Seurat using the Louvain algorithm modularity optimization. In all datasets, to identify all pancreatic endocrine and exocrine cells we identify the expression levels of the canonical pancreatic cell type markers: including INS (beta-cells), GCG (alpha-cells), SST (delta-cells), PPY (PP-cells), GHRL (epsilon-cells), PRSS1 (acinar cells), KRT19 (duct cells), COL1A1 (pancreatic stellate cells PSC/mesenchymal)) and ESAM (endothelial cells).

#### Differential gene expression, pathway, and gene ontology analysis

Differential expression gene (DEG) was performed to identify the top differentially overrepresented in each treatment, cell type, or cell subpopulation using the Wilcoxon Rank Sum Tes applying the Bonferroni correction to calculate the adjusted p-value. The gene list from the DEG analysis was used to perform a gene and pathway analysis using the clusterProfile package from R (18).

## Results

### Single-cell transcriptomics of primary human islet cells exposed to cytokine stress

**Figure 1:**
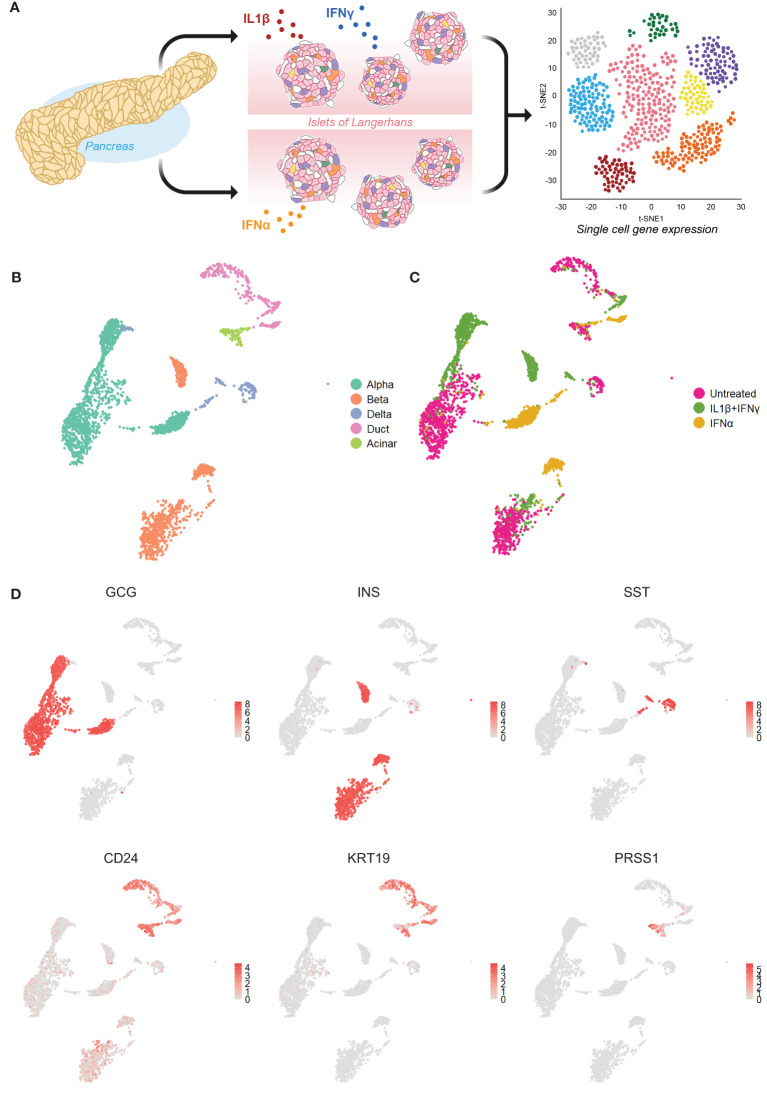
Single-cell RNA sequencing of primary human islet cells exposed to cytokine stress. **(A)** Schematic representation of the experimental set-up used to generate our single-cell RNA sequencing dataset from primary human pancreatic islet cells (n=3) exposed to IL1β+IFNγ or IFNα, or left untreated. **(B, C)** UMAP visualization of the main pancreatic cell types **(B)** and cytokine treatments **(C)**. **(D)** UMAP plots showing the average expression levels of canonical markers for alpha (glucagon – GCG), beta (insulin - INS), delta (somatostatin – SST), exocrine (CD24) including duct (keratin 19 - KRT19) and acinar (trypsin 1 - PRSS1) cells.

We first set out to investigate the molecular response of the main pancreatic cell types in response to inflammation by mining our single-cell transcriptomics dataset (GSE218316) of primary human islet cells subjected to IL1β+IFNγ or IFNα or left untreated (**Figure 1A**). We identified the main endocrine cell types by the expression of the canonical identity markers: INS (beta cells), GCG (alpha cells), and SST (delta). The main exocrine pancreatic cells (CD24) were identified by the expression of KRT19 (duct cells) and PRSS1 (acinar cells) (**Figures 1B-D**).

The donor batch effect did not influence the cell clustering (data not shown). However, the effect of cytokine treatment affected considerably the gene expression profiles, leading to the formation of separate clusters for each pancreatic cell type (**Figures 1B, C**; **Supplementary Figure 1A**). In subsequent analyses, we focused on alpha, beta, and duct cells, primarily as delta and acinar cells display an overall lower number of cells (**Supplementary Figure 1B**).

### Primary human duct cells display a pro-inflammatory profile upon *in vitro* inflammatory conditions

**Figure 2:**
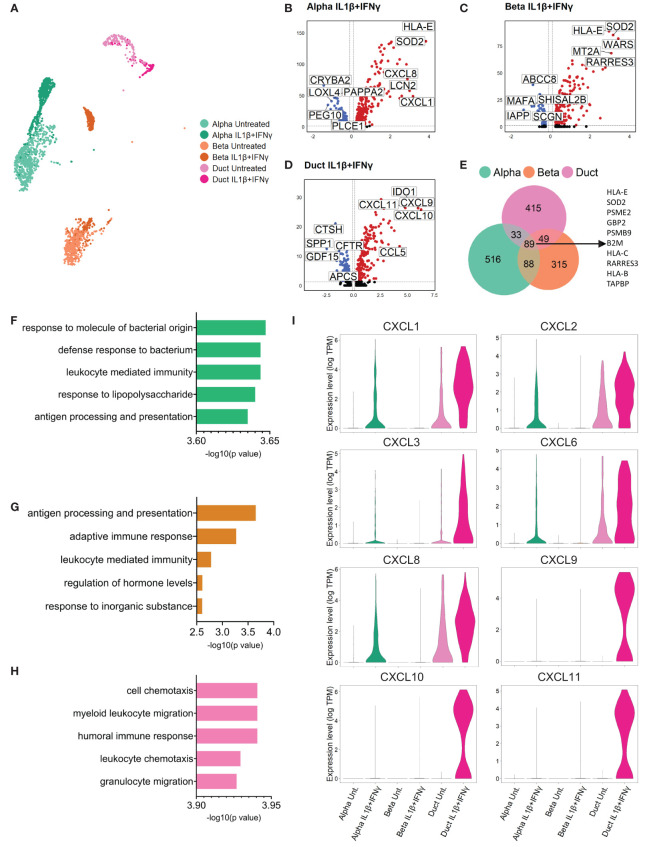
Primary human duct cells display a pro-inflammatory profile upon IL1β+IFNγ treatment. **(A)** UMAP plot showing sub-clusters of untreated and IL1β+IFNγ-treated pancreatic alpha, beta, and duct cells. **(B-D)** Volcano plots of the significant (adjusted p-value < 0.05) down-regulated (log 2-fold change < -0.1; blue) and up-regulated (log 2-fold change > 0.1; red) genes upon IL1β+IFNγ treatment in alpha, beta, and duct cells. **(E)** Venn diagrams of the differentially expressed genes (adjusted p-value <0.05) in alpha, beta, and duct cells treated upon IL1β+IFNγ treatment. **(F-H)** Bar plots showing the significance of the top 5 gene ontology terms from the GSE analysis performed in alpha **(F)**, beta, **(G)**, and duct **(H)** cells treated with IL1β+IFNγ. **(I)** Violin plots showing the average gene expression in transcripts per million (TPM, log scale) of pro-inflammatory-related genes in alpha, beta, and duct cells treated with IL1β+IFNγ.

We performed a differential gene expression analysis to identify the top up- and down-regulated genes by comparing each cytokine condition to the untreated group in every cell type (**Figure 2** (IL1β+IFNγ), **Supplementary Figure 1** (IFNα), **Supplementary Tables 2–4**). Differential expression analysis performed in cells treated with the combination of IL1β+IFNγ revealed that alpha, beta, and duct cells commonly expressed genes involved in antigen processing and presentation, including PSME2, PSMB9, TAPBP, HLA-B, C, and E (**Figures 2B-E**). Furthermore, gene set enrichment analysis (GSEA) revealed that the top biological processes activated by IL1β+IFNγ in duct cells are associated with immune cell recruitment (leukocyte, neutrophil, and granulocyte chemotaxis), and this is not the case in alpha or beta cells (**Figures 2F-H**).

The genes present in these gene ontology terms were mostly from the chemokine (C-X-C motif) ligand family (CXCL) (**Figure 2I**, **Supplementary Tables 5, 6**). Duct cells showed the most prominent upregulation of CXCL-1, 2, 3, and 6 as compared to alpha and beta cells. CXCL8 was found to be up-regulated in both endocrine and exocrine pancreatic cells. Finally, CXCL-9, 10, and 11 were only expressed and induced in duct cells upon IL1β+IFNγ treatment (**Figure 2I**, **Supplementary Figure 1J**).

IFNα promoted specific interferon-related pathways in duct cells, but not in alpha and beta cells (**Supplementary Figures 1G-I**). However, IFNα treatment did not induce upregulation of CXCL genes in any pancreatic cell type (**Supplementary Figure 1J**).

Collectively these data show that pancreatic duct cells specifically respond to inflammation by upregulating a set of pro-inflammatory chemokines that are associated with immune cell recruitment and activation, positive regulation of the immune effector process, and regulation of T cell proliferation associated with immune cell activation.

### Primary human duct cells display a pro-inflammatory profile under *in vivo* conditions associated with inflammation

We then asked whether the pro-inflammatory phenotype of duct cells could also be seen in pathophysiological conditions. We used a single-cell RNA sequencing dataset we generated from pancreatic cells obtained from donors with a history of T1D (n=2, **Supplementary Figures 2A, B**). We found that among all the main pancreatic cell types, duct cells are again the major source of pro-inflammatory cytokines, including CXCL1, CXCL2, CXCL3, and CXCL8, while CXCL9, 10, and 11 are not expressed (**Supplementary Figure 2C**).

Of note, CXCL8 was also expressed in endocrine cells, as seen in the IL1β+IFNγ treated cells (**Supplementary Figure 1**). We asked whether CXCL8 could affect the beta cells. Treatment of primary human islets with recombinant CXCL8 for 24 hours showed a trend towards a reduction in beta cell function (**Supplementary Figure 2D**). Furthermore, human islets treated with an anti-CXCL8 blocking antibody showed better insulin secretion upon glucose stimulus under cytokine treatment (**Supplementary Figure 2E**). This indicates that, aside from their chemoattractant role, chemokines may also have a detrimental effect on beta cell health.

Finally, we had the opportunity to process single-cell RNA-sequencing pancreatic cells from an individual diagnosed with Wolfram Syndrome (n=1, **Supplementary Figures 2F, G**). There as well, we observed a specific upregulation of pro-inflammatory cytokines CXCL1, CXCL2, and CXCL8 in the duct cells (**Supplementary Figure 2H**). Of note, this dataset was unfortunately very poor in endocrine cells. But, overall, this data confirmed that duct cells are particularly prone to developing an enhanced pro-inflammatory profile in pancreatic diseases.

### Duct-acinar cells are the main subpopulation of duct cells displaying a pro-inflammatory signature

**Figure 3:**
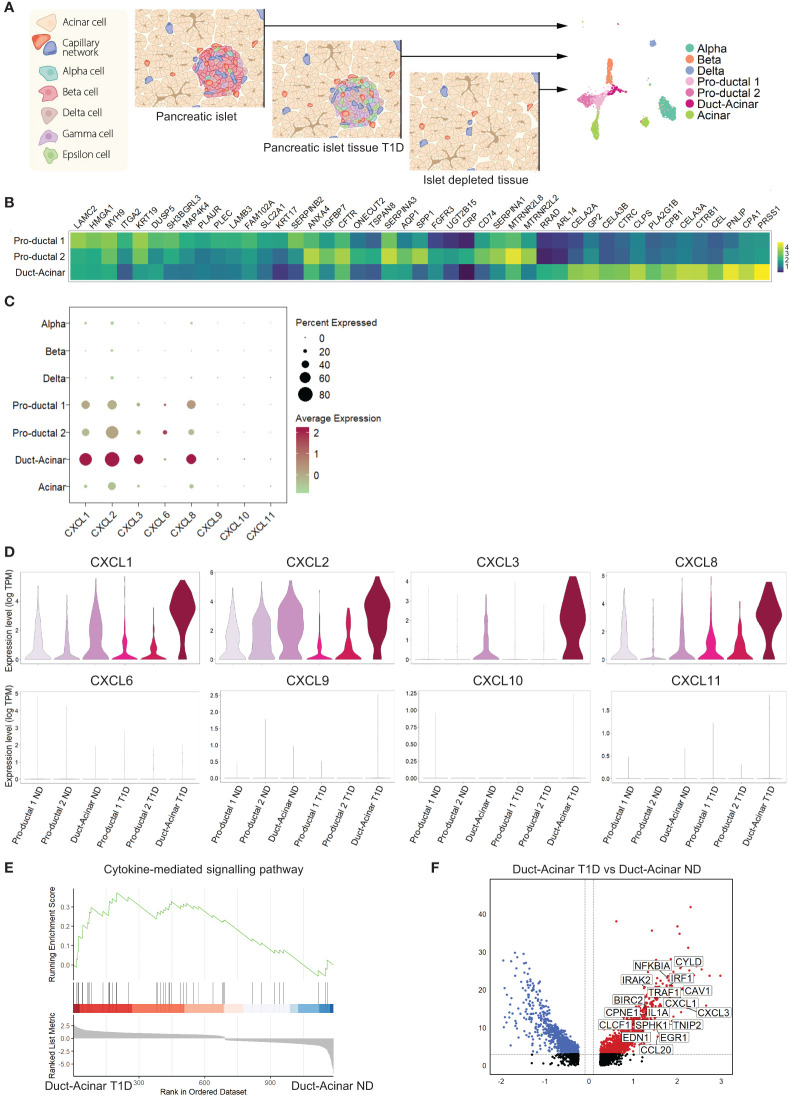
Duct-acinar cells are the main subpopulation of duct cells displaying a pro-inflammatory signature. **(A)** Schematic representation of the integration of single-cell RNA-sequencing datasets from pancreatic islets obtained from organ donors without diabetes (n=3), pancreatic cells from organ donors with T1D (n=2), and cells from islet-depleted tissue from 4 other ND donors (n=4). **(B)** Heatmap of the top 15 overrepresented genes in each duct cell subpopulation when compared to each other. **(C)** Dot plot showing the relative expression of pro-inflammatory cytokines in all cluster cell types groups. **(D)** Violin plots showing the average gene expression in transcripts per million (TPM, log scale) of pro-inflammatory genes in the different duct cell subpopulations from donors without diabetes (ND) or type 1 diabetes (T1D). **(E)** Plot showing running enrichment score of the cytokine-mediated signaling pathway in duct-acinar cells comparing T1D from ND. **(F)** Volcano plot showing the significantly (adjusted p-value < 0.05) down-regulated (log 2-fold change < -0.1) and up-regulated (log 2-fold change > 0.1) genes in duct-acinar cells comparing T1D and ND. Genes from the cytokine-mediated signaling pathway gene ontology term are shown.

To further characterize the response of primary human duct cells to inflammation, we integrated single-cell RNA-sequencing datasets from islet cells obtained from organ donors without diabetes (ND) (n=3), pancreatic cells from organ donors with T1D (n=2) donors, and cells from islet-depleted tissue from 4 other ND donors (**Figure 3A**). For further analysis, we decided not to include the Wolfram dataset in our integration due to the batch effect (only one donor was included and the sequencing platform was different). Cell integration and clustering resulted in the identification of the different endocrine and exocrine pancreatic cell types, including three duct subpopulations (**Supplementary Figures 3A–C**). The labelling of these three duct cell subpopulations as ‘pro-ductal 1’ that express typical small duct cell markers (KRT19^high^, KRT17^high^), ‘pro-ductal 2’ that expresses genes related to duct progenitor markers (SPP1, CRP, TSPAN8), and ‘duct-acinar’ that expresses genes from acinar-like cells (CLPS, CEL, CELA2A/3A/3B) was based on their top overrepresented genes, when duct cell sub-populations were compared between each other, and the classification proposed by Qadir et al. (19) (**Figure 3B**).

When focusing on the expression of the pro-inflammatory cytokines reported in our *in vitro* study (**Figure 2I**), we found that duct cells, and more specifically cells from the duct-acinar subpopulation, express CXCL1, 2, 3, and 8 (**Figure 3C**). Interestingly, the expression of CXCL1, 2, and 3 was more upregulated in the duct-acinar subpopulation in T1D as compared to ND (**Figure 3D**). Furthermore, gene set enrichment analysis revealed that duct-acinar cells presented enrichment in genes involved in cytokine-mediated signaling (**Figure 3E**) as compared to the rest of the duct cell subpopulations in T1D (**Supplementary Table 7**), including CXCL1 and 3 and IL1α (**Figure 3F**).

**Figure 4:**
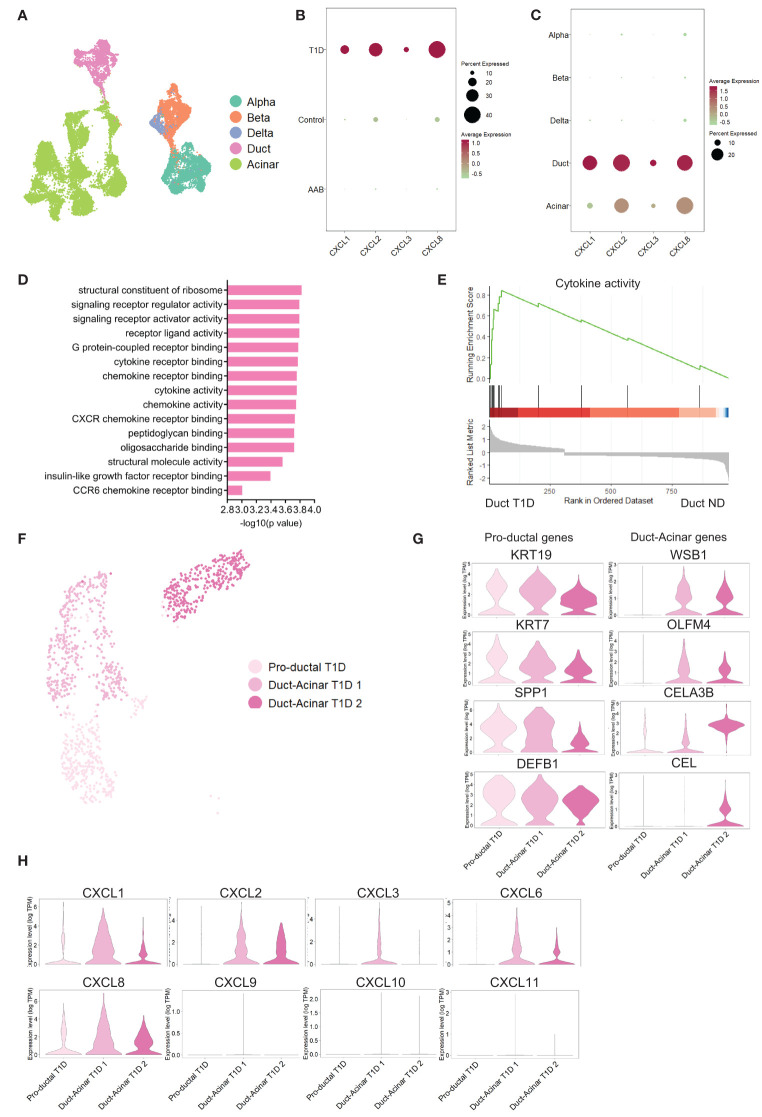
Validation of the findings in an independent dataset from pancreatic tissue from T1D individuals. **(A)** UMAP plot of the single-cell RNA-sequencing dataset retrieved from the Fasolino et al. (20) study. **(B, C)** Dot plots of the relative expression of pro-inflammatory cytokines in clustered cells based on disease and cell type background respectively. **(D)** Bar plot visualization of the most significant gene ontology terms in the GSE analysis performed in T1D duct cells versus ND duct cells. **(E)** Plot showing running enrichment score of cytokine activity gene ontology term in T1D duct cells compared to ND duct cells. **(F)** UMAP plot of pancreatic cell subpopulations identified in T1D cells. **(G)** Violin plots showing the average gene expression in transcripts per million (TPM, log scale) from the proposed duct subpopulations markers from Qadir et al. (19). **(H)** Violin plots showing pro-inflammatory gene expression in the different duct cell subpopulations.

To validate these findings in an independent dataset, we retrieved the publicly available dataset from Fasolino et al. (20) that reports a single-cell RNA sequencing study of islet cells from individuals with T1D (n=5), individuals positive for beta cell antibodies (AAbs) but not diagnosed with T1D (n=8) and ND individuals (n=8) (**Figure 4A**). Analysis of this dataset showed a higher pro-inflammatory transcriptional signature in pancreatic cells from T1D donors (**Figure 4B**). Furthermore, among all pancreatic cell types, duct cells were again the major source of pro-inflammatory genes (**Figure 4C**). Gene set enrichment analysis revealed that duct cells from T1D donors showed enrichment in cytokine-related processes when compared to duct cells from ND donors (**Figures 4D, E**). The enrichment of these pro-inflammatory pathways was specific to T1D duct cells as compared to all other pancreatic cell types (**Supplementary Figures 4A–D**; **Supplementary Table 8**). Finally, the subclustering of duct cells from T1D confirmed the presence of three duct cell subpopulations (**Figure 4F**). Similar to our own dataset, we identified the 3 ductal subclusters based on Qadir et al. (19): pro-ductal that express KRT19^high^, KRT7^high^, SPP1^high,^ and DEFB^1high^ (**Figure 4G**) and no expression of acinar-like markers; in contrast, duct-acinar 1 and 2 express intermediate and high levels of acinar-like markers, respectively (**Supplementary Figures 4E, F**). In addition, we further confirmed that duct-acinar 1 cells express WSB1 and OLFM4^high^, and duct-acinar cells 2 express WSB1, CELA3B^high^, and CEL (**Figure 4G**). Finally, we confirmed that the expression of pro-inflammatory chemokines is mainly expressed in duct-acinar subpopulations (**Figure 4H**).

## Discussion

Islet inflammation is characteristic of most types of diabetes. There is growing evidence that inflammation is also present in the exocrine compartment (21, 22). However, little is known about the potential implication of exocrine cell types in the development and/or progression of islet inflammation. Here we show that a subpopulation of duct cells, so-called duct-acinar cells, respond to inflammation by strongly upregulating a set of pro-inflammatory chemokines that may contribute to the recruitment and activation of immune cells to the pancreatic islet niche.

Previous studies have shown an increased expression of pro-inflammatory chemokines from the CXC and CXCL motif family genes as a response to inflammation stimuli within the pancreatic islets (23). In addition, a correlation has been established between the expression of CXCL-related chemokines, immune cell infiltration into the pancreatic islet, and the development of diabetes (23–25). However, none of these studies have investigated the potential role of the other pancreatic cell types.

We identified a set of pro-inflammatory cytokines that may be used as biomarkers for the onset and progression of pancreatic inflammation in diabetes. We found that, under inflammation, pancreatic duct cells developed an exclusive and characteristic phenotype that involved the production of pro-inflammatory cytokines, including CXCL-1, -2, -3, -9, -10, and -11. Interestingly, CXCL-9, -10, and -11 have been defined as a determinant axis to promote immune cell recruitment and activation (26), and have been suggested to be the main cause of autoimmune diseases (27). Previous work has proposed CXCL-9 and CXCL-10 as potential biomarkers of type 1 diabetes mellitus (28). In addition, in the same study, the authors demonstrated that CXCL1 is also increased in the same patients with the disease. In line with these observations, we observe that, under inflammation, the co-expression of CXCL-9, 10, and -11 (along with CXCL1, -2, and -3) is increased in the pancreatic tissue. Importantly, we show that this response is exclusively occurring in duct cells.

In addition, we found that higher levels of chemokines are expressed in T1D, as compared to cells from ND donors, and again mainly in duct cells. Moreover, it was interesting to observe that duct cells from an individual with Wolfram syndrome also showed a specific increase in the level of pro-inflammatory chemokines. Although known as a genetic disease in which endoplasmic dysfunction is considered to be the main cause of beta cell death (29), it has been reported that mutations in the WFS1 gene that are specific to this disease have been associated with increased systemic inflammation (3). These findings are in line with the observations we made in the T1D and cytokine treatment datasets, yet the analysis remains limited to pancreatic cells from only one donor.

Recent studies have suggested that the exocrine pancreas, more specifically duct cells, have an important role in the pathogenesis of T1D (30). Our study not only revealed a potential major implication of duct cells in immune cell recruitment and activation, but it also sheds light on the heterogeneity of duct cell subpopulations in the modulation of inflammation. Previous single-cell RNA sequencing studies revealed that duct cells that are positive for CELA2A, CLPS, CEL, and CPA1 are considered duct-acinar cells (19). Our data shows that duct-acinar cells are the main source of pro-inflammatory cytokines, suggesting that they play a crucial role in the recruitment and activation of immune cells in the pancreas. We were able to investigate duct cells from T1D patients using a large-scale scRNAseq dataset from Fasolino et al. (20). In this dataset, based on Qadir duct cell subpopulation classification, we could identify one pro-ductal and two duct-acinar subpopulations; confirming that the expression of pro-inflammatory molecules was enriched in the duct-acinar populations. In line with Fasolino et al. (20) observations, we report that the duct-acinar subpopulation expresses, although at low levels, ICAM1, ISG20, and IRF7, which are essential for immune cell interaction and the promotion of inflammation (31–33).

Overall, the use of the powerful single-cell RNA sequencing technology allowed us to investigate the heterogeneity of the pancreatic ductal cells. The findings, though based on mRNA analyses exclusively, point towards a major role of duct cells in the amplification of inflammation in diabetes. Therefore, more efforts are needed to further elucidate the role of the different pancreatic duct subpopulations in the context of islet inflammation in diabetes.

## Data availability statement

Single-cell RNA-seq data is available in the Gene Expression Omnibus (GEO) and reachable with the following accession numbers [GSE218316, GSE 263365, GSE263364, GSE263565].

## Ethics statement

Human islets were used for research only if they could not be used for clinical purposes, and if research consent was present, according to Dutch national laws. The studies were conducted in accordance with the local legislation and institutional requirements. The human samples used in this study were acquired from a by-product of routine care or industry. Written informed consent to participate in this study was not required from the participants or the participants’ legal guardians/next of kin in accordance with the national legislation and the institutional requirements.

## Author contributions

AM: Conceptualization, Formal analysis, Investigation, Methodology, Writing – original draft, Writing – review & editing. JJ: Formal analysis, Investigation, Writing – original draft, Writing – review & editing. NG: Formal analysis, Methodology, Writing – original draft, Writing – review & editing. AZ: Conceptualization, Formal analysis, Writing – original draft, Writing – review & editing. EK: Conceptualization, Funding acquisition, Resources, Supervision, Writing – original draft, Writing – review & editing. FC: Conceptualization, Formal analysis, Investigation, Methodology, Writing – original draft, Funding acquisition, Project administration, Resources.
