# Supplementary material for: Single-cell transcriptomics reveals a role for pancreatic duct cells as potential mediators of inflammation in diabetes mellitus
Source: Front Immunol. 2024 Apr 29;15:1381319. doi: 10.3389/fimmu.2024.1381319 (PMC11089191; doi:10.3389/fimmu.2024.1381319)
Supplement: Supplementary file 1 [file DataSheet_1.docx]

**Supplementary Fig. 1. Primary human duct cells display a distinct pro-inflammatory profile upon IL1β+IFNγ or IFNα treatment.** (A) UMAP plot of all endocrine and exocrine pancreatic cells from the pancreatic islets exposed to inflammatory stress dataset (GSE218316) including cells from untreated, the combination of IL1β+IFNγ and the single treatment of IFNα. (B) Bar plot of the number of cells treated by each stressor per cell type. (C to E) Volcano plots showing the significant (adjusted p-value < 0.05) down-regulated (log 2-fold change < -0.1) and up-regulated (log 2-fold change > 0.1) upon IFNα treatment in alpha, beta, and duct cells. (F) Venn diagrams resulting from the comparison of the gene list containing the genes significantly altered (adjusted p-value <0.05) in alpha, beta, and duct cells treated with the single treatment of IFNα. (G to I) Bar plots showing the significance of gene ontology terms from the GSE analysis performed in alpha (G), beta (H), and duct (I) cells treated with IFNα. (J) Violin plots representing average gene expression of the pro-inflammatory chemokines in the different endocrine and exocrine cells exposed to the single treatment of IFNα and the combination of IL1β+IFNγ.

**Supplementary Fig. 2. Single-cell RNA sequencing approach performed in human pancreatic islet from T1D and Wolfram syndrome patients.** (A) UMAP plot of the pancreatic cell types detected in the T1D dataset (B) UMAP visualization representing the expression levels of pro-inflammatory cytokines in the different pancreatic cell clusters. (C) Violin plot showing normalized log 2 count values of pro-inflammatory cytokine-related genes in each pancreatic cell type. (D and E) Bar plot showing the stimulation index representing insulin secretion upon glucose stimulus in human pancreatic islets treated with CXCL8 and human pancreatic islets treated with cytokines (IL1β+IFNγ) and cytokines with a CXCL8-blocking antibody respectively. (F) UMAP visualization of the different pancreatic cell types detected in the Wolfram syndrome dataset. (G) UMAP plot showing the expression levels of pro-inflammatory cytokines in the different pancreatic cell clusters. (H) Violin plot showing normalized log 2 count values of pro-inflammatory cytokines in each pancreatic cell type.

**Supplementary Fig. 3. Related to main Fig. 3.** (A) UMAP maps of the average expression levels of canonical markers to identify: alpha (glucagon – GCG), beta (insulin - INS), delta (somatostatin – SST), (PPY, pancreatic polypeptide) gamma, exocrine (CD24) including duct (keratin 19 - KRT19 and CFTR) and acinar (trypsin 1 - PRSS1) cells. (B) UMAP plot of the cell type information per cell. Pie chart representing the number of pancreatic cell types in the dataset. (C) UMAP plot showing donor information per cell. (D) Bar plot representing pancreatic cell types in the dataset grouped by donor. (E) Bar plot of the significance of gene ontology terms from the GSE analysis performed in T1D duct-acinar cells compared to ND duct-acinar.

**Supplementary Fig. 4. Related to main Fig. 4.** (A) UMAP plot of the pancreatic cells from T1D donors extracted from Fasolino’s dataset [15]. (B, C, D) Bar plot showing the significance of gene ontology terms from the GSE analysis performed in T1D compared to ND pancreatic cells (alpha, acinar, and beta respectively – GSEA performed in delta cells showed no enriched GO terms). (E, F) Heatmaps of the average expression of acinar-like markers and tolerogenic dendritic cell markers mentioned in Fasolino et al. [15] respectively.
