# Supplementary material for: Single-cell transcriptomics reveals a role for pancreatic duct cells as potential mediators of inflammation in diabetes mellitus
Source: Front Immunol. 2024 Apr 29;15:1381319. doi: 10.3389/fimmu.2024.1381319 (PMC11089191; doi:10.3389/fimmu.2024.1381319)
Supplement: Supplementary file 2 [file Presentation_1.pptx]

## Slide 1
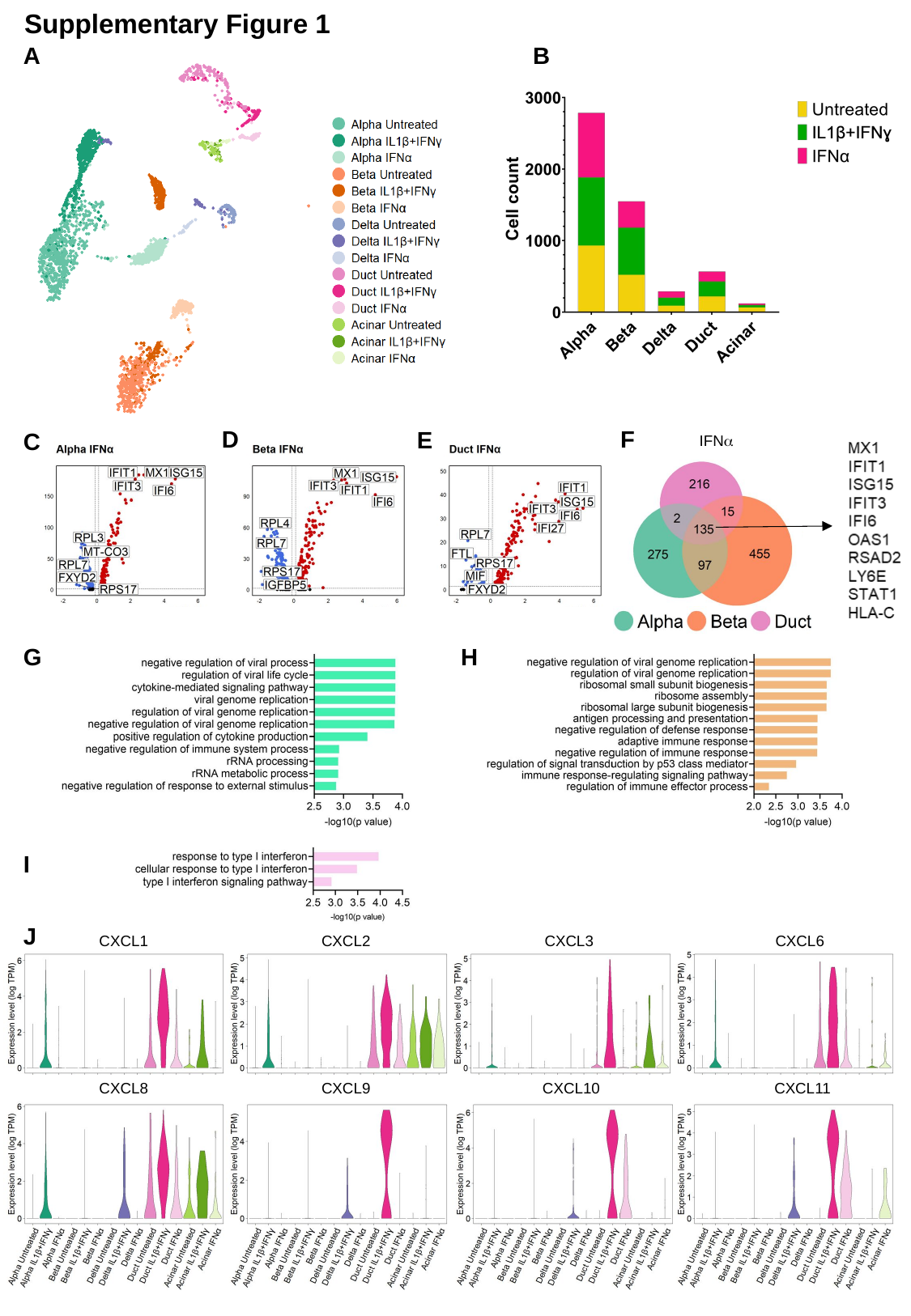

Supplementary Figure 1
A
B
D
F
E
C
IFNa
G
H
I
J
CXCL1
CXCL2
CXCL3
CXCL6
CXCL8
CXCL9
CXCL10
CXCL11

## Slide 2
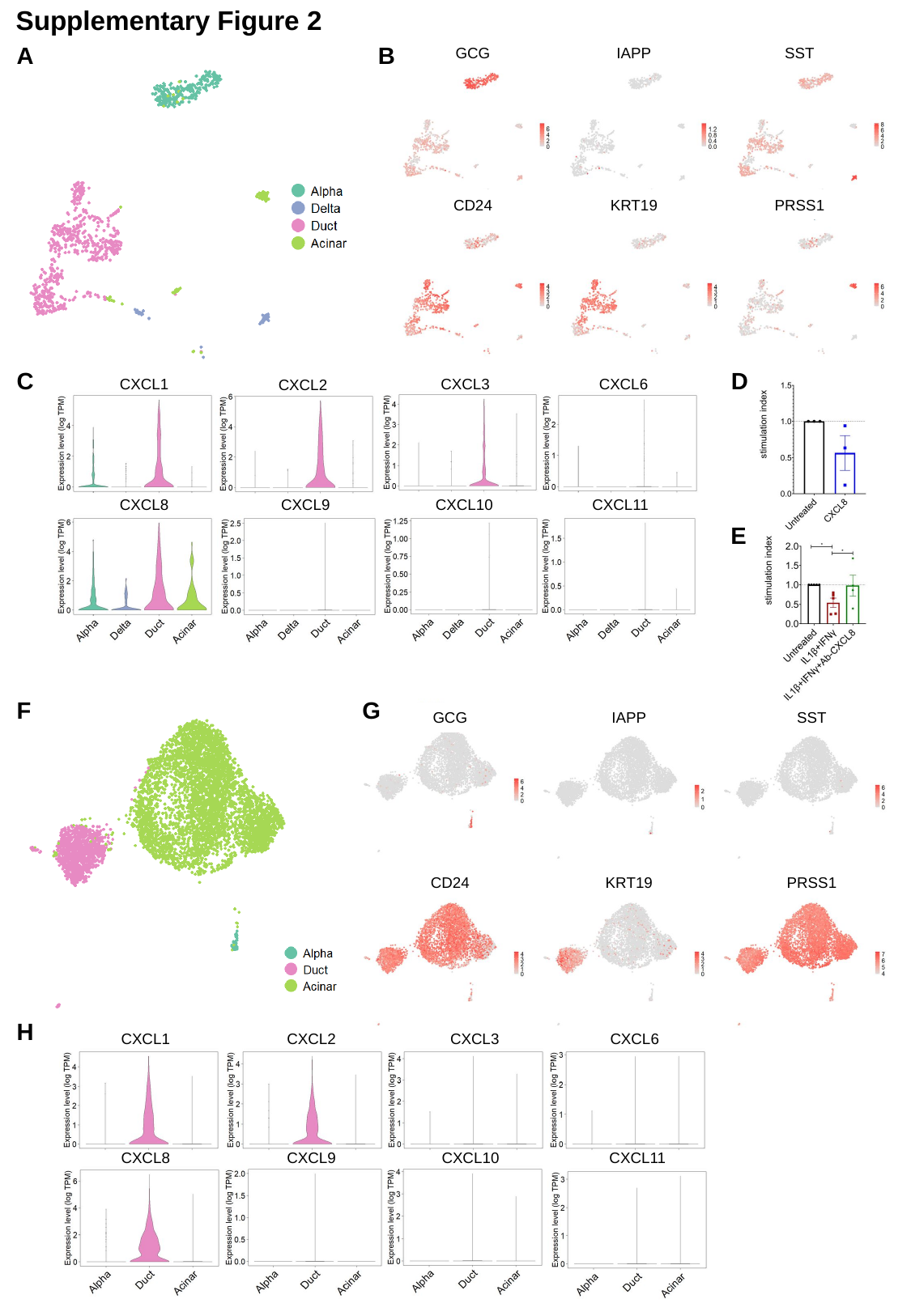

Supplementary Figure 2
A
B
GCG
IAPP
SST
CD24
KRT19
PRSS1
C
D
CXCL1
CXCL3
CXCL6
CXCL2
CXCL8
CXCL9
CXCL10
CXCL11
E
F
G
GCG
IAPP
SST
CD24
KRT19
PRSS1
H
CXCL1
CXCL2
CXCL3
CXCL6
CXCL8
CXCL9
CXCL10
CXCL11

## Slide 3
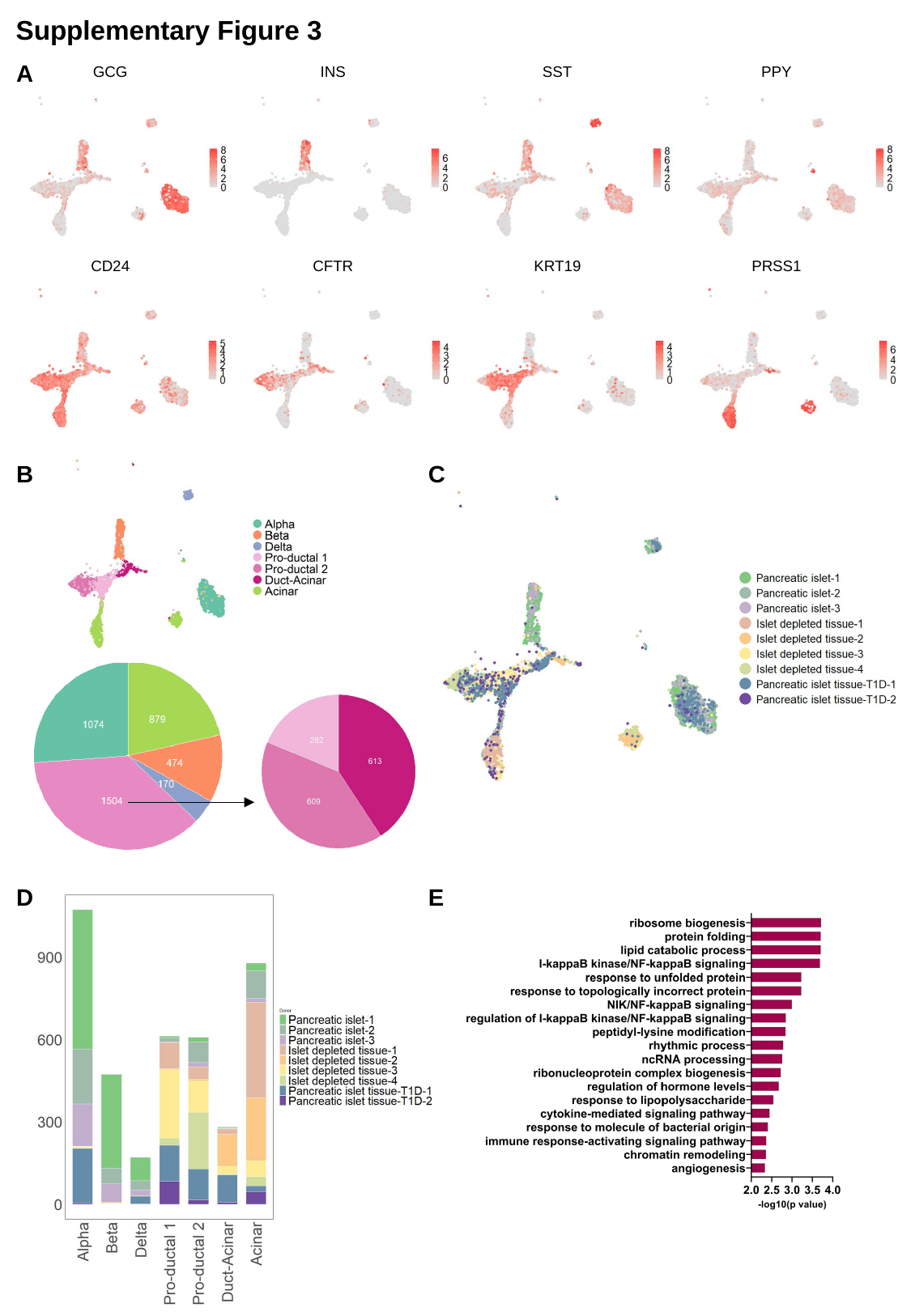

Supplementary Figure 3
A
GCG
INS
SST
PPY
CD24
CFTR
KRT19
PRSS1
B
C
D
E

## Slide 4
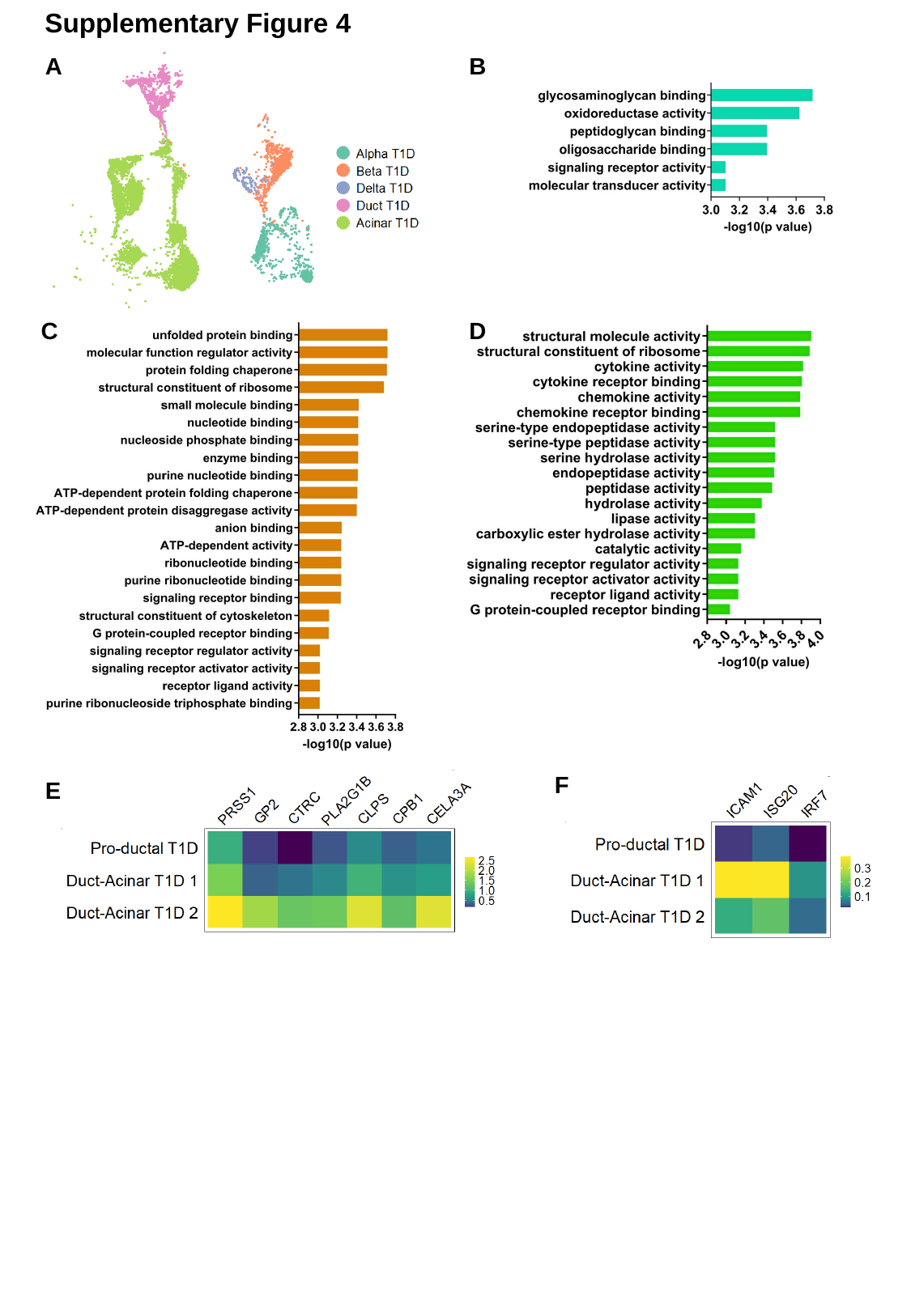

Supplementary Figure 4
A
B
C
D
F
E
