# Supplementary material for: Single-cell transcriptomics reveals a role for pancreatic duct cells as potential mediators of inflammation in diabetes mellitus
Source: Front Immunol. 2024 Apr 29;15:1381319. doi: 10.3389/fimmu.2024.1381319 (PMC11089191; doi:10.3389/fimmu.2024.1381319)
Supplement: Supplementary file 3 [file Table_1.docx]

**Supplementary Table 1**: **Donor information related to the samples used in this study:**

Adapted from Hart NJ, Powers AC (2018) Progress, challenges, and suggestions for using human islets to understand islet biology and human diabetes. Diabetologia <https://doi.org/10.1007/s00125-018-4772-2>.

| **Preparation** | **1** | **2** | **3** | **4** | **5** | **6** | **7** |  |
| --- | --- | --- | --- | --- | --- | --- | --- | --- |
|  | | | | | | | | |
| Unique identifier | R106 | P479 | P570 | P575 | P578 | P587 | R138 |  |
| Donor age range (years) | 50-54 | 55-59 | 45-49 | 50-54 | 15-19 | 55-59 | 75-79 |  |
| Donor sex (M/F) | M | F | F | F | F | F | M |  |
| Donor BMI (kg/m^2^) | 26 | 19 | 22 | 21 | 22 | 24 | 22 |  |
| Donor HbA_1c_ or other measure of blood glucose control | n.a. | n.a. | 5.5* | 5.4* | 5.5* | 5.6* | n.a. |  |
| Origin/source of islets^b^ | LUMC | LUMC | LUMC | LUMC | LUMC | LUMC | LUMC |  |
| Islet isolation centre | LUMC | LUMC | LUMC | LUMC | LUMC | LUMC | LUMC |  |
| Donor history of diabetes? Yes/No | No | No | No | No | No | No | No |  |
| **If Yes, complete the next two lines if this information is available** | | | | | | | | |
| Diabetes duration (years) |  |  |  |  |  |  |  |  |
| Glucose-lowering therapy at time of death^c^ |  |  |  |  |  |  |  |  |

*last glucose measurement at ICU

n.a. not available

| **Preparation** | **8** | **9** | **10** | **11** | **12** | **13** | **14** | **15** |
| --- | --- | --- | --- | --- | --- | --- | --- | --- |
|  | | | | | | | | |
| Unique identifier | SAMN10490796 | SAMN12673306 | SAMN13319813 | R124 (T1D) | R141 (T1D) | R154 (T1D) | R228 (WS) | R102 |
| Donor age (years) | 50-54 | 55-59 | 55-59 | 45-49 | 50-55 | 20-24 | 50-54 | 50-54 |
| Donor sex (M/F) | F | M | M | M | M | F | M | M |
| Donor BMI (kg/m^2^) | 26.4 | 28.4 | 26.7 | 28 | 23 | 30 | 25 | 25 |
| Donor HbA_1c_ or other measure of blood glucose control | 5.4 | 5.6 | 6.1 | 6.7* | 14.6* | 13.9* | n.a. | n.a. |
| Origin/source of islets^b^ | IIDP | IIDP | IIDP | LUMC | LUMC | LUMC | LUMC | LUMC |
| Islet isolation centre | The Scharp-Lacy Research Institute | The Scharp-Lacy Research Institute | University of Miami | LUMC | LUMC | LUMC | LUMC | LUMC |
| Donor history of diabetes? Yes/No | No | No | No | Yes | Yes | Yes | Yes | No |
| **If Yes, complete the next two lines if this information is available** | | | | | | | | |
| Diabetes duration (years) |  |  |  | 13 years | n.a. | 14 years | 41 years |  |
| Glucose-lowering therapy at time of death^c^ |  |  |  | n.a. | n.a. | n.a. | n.a. |  |

*last glucose measurement at ICU

n.a. not available
